# Silent but significant: Functional elucidation of a synonymous ATP7B mutation in Wilson’s disease pedigrees

**DOI:** 10.3389/fgene.2025.1604683

**Published:** 2025-06-10

**Authors:** Qi Zhang, Xiaoming Xie, Hairong Li, Yulei Li, Hongyao Huang

**Affiliations:** ^1^ Jinzhou Medical University Graduate Training Base, Suizhou Central Hospital Affiliated to Hubei University of Medicine, Suizhou, Hubei, China; ^2^ Xiangyang Integrated Traditional Chinese and Western Medicine Hospital, Xiangyang, Hubei, China; ^3^ Respiratory Endoscopy Center, Suizhou Central Hospital, Hubei University of Medicine, Suizhou, Hubei, China; ^4^ Xiangyang Central Hospital, Affiliated Hospital of Hubei University of Arts and Science, Xiangyang, China; ^5^ Department of Laboratory, Suizhou Central Hospital, Hubei University of Medicine, Suizhou, Hubei, China

**Keywords:** degeneration, ATP7B gene, synonymous mutation, Sanger sequencing, minigene assay

## Abstract

**Introduction:**

Wilson’s disease (hepatolenticular degeneration) is a common hereditary neurological disorder. Early diagnosis, particularly the widespread implementation of genetic testing and timely intervention, is crucial for improving the prognosis of this disease. However, limited data exist on genotype-phenotype correlations, thereby impeding accurate early clinical diagnosis.

**Methods:**

Whole-exome sequencing was performed on the proband and family members to detect genetic variants associated with the clinical phenotype. Bioinformatics tools (HSF, SpliceAI and ESEfinder 3.0) were used to predict the impact of mutations on the splicing function of precursor mRNA. The *in vitro* minigene experiment was conducted to verify the impact of the mutation on the splicing function of the precursor mRNA.

**Results:**

Whole-exome sequencing of the proband identified a synonymous variant c.2145C>T (p. Tyr715=) and a pathogenic frameshift mutation c.2304dupC (p. Met769Hisfs*26) in the *ATP7B* gene, both associated with the clinical phenotype. The frameshift mutation c.2304dupC (p. Met769His fs*26) on the other allele was a known pathogenic variant causing protein truncation. Bioinformatics tools (HSF, SpliceAI, and ESEfinder 3.0) predicted that the c.2145C>T mutation might disrupt nearby splicing sites. *In vitro* minigene assays confirmed aberrant precursor mRNA splicing caused by *ATP7B* c.2145C>T (p. Tyr715=) synonymous mutation, resulting in reduced abundance of normal transcripts.

**Conclusion:**

The compound heterozygous variants (c.2145C>T and c.2304dupC) in ATP7B likely synergistically contribute to the proband’s abnormal clinical phenotype, aligning with the recessive inheritance pattern of Wilson’s disease.

## Graphical Abstract

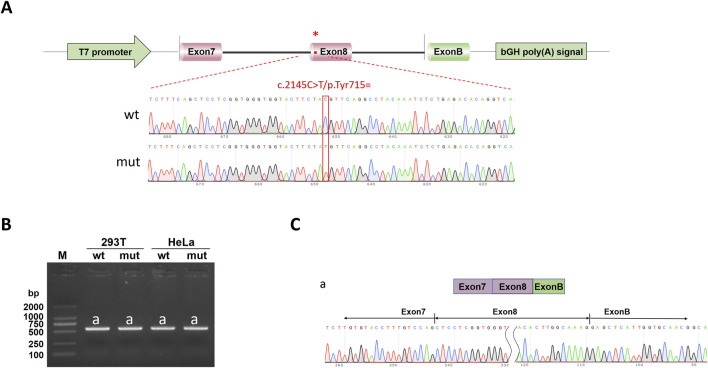

## 1 Introduction

Hepatolenticular degeneration (OMIM: 277900), also known as Wilson’s disease (WD), is an autosomal recessive genetic disease mainly characterized by abnormal copper metabolism caused by ATP7B gene mutation (Huang and Liu, 2019), which mainly involves the liver, brain, and cornea. The disease usually occurs in childhood and adolescence, and it is clinically manifested as mental symptoms such as emotional instability, distraction, and memory loss, or neurological symptoms dominated by dance-like movements and muscular dystonia (Kodama et al., 2012). The ATP7B gene, which causes hepatolenticular degeneration, is located on human chromosome 13 and contains 21 exons, encoding a protein that is a member of the P-type copper-transporting ATPase family (Hartwig et al., 2019). This protein has the function of copper transport ATPase, which can expel copper out of the cell. When mutations occur in the ATP7B gene, the function of the encoded protease decreases or is lost, leading to a reduction in the synthesis of serum ceruloplasmin and subsequently causing impaired excretion of copper in the blood and bile ducts (Wang et al., 2019). Excessive accumulation of copper could lead to cell necrosis and, in severe cases, it could cause dysfunction of organs such as the brain, liver, and kidneys and may even result in the death of patients. Mutations in the ATP7B gene are diverse and can occur anywhere within the gene, including the promoter region, exon regions, and even intron regions (Wang et al., 2019). The specific mechanisms underlying ATP7B gene mutations and the pathogenesis of Wilson’s disease remain incompletely elucidated.

A Wilson’s disease proband and parental pedigree is reported in this study, in which whole-exome sequencing (WES) identified compound heterozygous ATP7B mutations in the proband: a synonymous mutation c.2145C>T (p. Tyr715 =) and a pathogenic frameshift mutation c.2304dupC (p. Met769Hisfs*26). The c.2304dupC variant has been previously confirmed as disease causing (Lao and Le, 2024). Although synonymous mutations do not directly alter the protein structure, they may disrupt precursor mRNA splicing by modifying cis-acting elements or trans-acting factor binding sites (Love et al., 2023). To clarify the pathogenicity of the c.2145C>T variant, bioinformatics predictions (HSF, SpliceAI, and ESEfinder 3.0) and *in vitro* minigene assays were performed, demonstrating that this mutation reduces normal transcript abundance by interfering with mRNA splicing. Combined with the confirmed pathogenic frameshift mutation on the other allele, these findings expand the genetic spectrum of Wilson’s disease and provide mechanistic insights into its molecular pathogenesis.

## 2 Materials and methods

### 2.1 Experimental reagents and instruments

DNA polymerase were purchased from TaKaRa Corporation, item number R045A. The plasmid extraction kit was purchased from SIMGEN, No. 1005250. The DNA glue recovery kit was purchased from SIMGEN, item No. 2001250. TRIzol reagent was purchased from TaKaRa Company, item No. 9190. The cDNA synthesis reagent and qPCR mix were purchased from Yeasen. The TA cloning kit was purchased from Yeasen, No. 10907ES50. The transfection kit was purchased from Yeasen, item No. 40802ES03. The ultra-clean table is SW-CJ-2FD from the Sujing Antai Company, the pure water meter is Milli-Q from Millipore, the table centrifuge is 5424 from Eppendorf, and the horizontal electrophoresis meter is JY-SPCT from the Junyi Oriental Company.

### 2.2 Whole-exome sequencing

The study object was a family that met the diagnostic criteria of hepatolenticular disease. The members include the father, mother, proband, proband’s brother, and proband’s sister. WES and Sanger sequencing were performed on the proband and their siblings and parents.

Diagnostic basis (European Association for Study of Liver, 2012): childhood or adolescent onset, history and/or symptoms of liver disease (most common in children), extrapyramidal symptoms (more common in adolescent patients), corneal pigment ring, and a positive family history; copper biochemical tests were abnormal, including the decrease of serum ceruloplasmin, the decrease of serum copper content, the increase of urinary copper in 24 h, the significant increase of urinary copper in penicillamine stress test, and the high copper content in cultured skin fibrocytes.

The study subject in this article was admitted to the hospital presenting with symptoms including dysarthria, ataxia, and dysphagia. The copper biochemical findings were as follows: blood copper 2.98 μmol/L, copper oxidase 0.004u/L, ceruloplasmin 52.3 mg/L; EKG (electro-cardiography): sinus rhythm; K–F ring (+++); abdominal B-ultrasonography showed hepatocellular changes, splenomegaly, cholecystitis, gallbladder polyps, and hepatocellular nephropathy.

Detection of exon region, upstream and downstream intron variation within 20 bp, and insertion/deletion variation less than 50 bp was carried out using high-throughput next-generation sequencing based on liquid phase capture technology. The human genome DNA extracted from peripheral blood or DNA provided by the subject was interrupted by ultrasound, and the library was prepared. After that, the target area was captured using IDT xGen Exome Research Panel v1.0, and the variation was detected using a high-throughput sequencing platform.

### 2.3 Bioinformatics prediction

Input the mutation information of ATP7B into HSF (https://www.genomnis.com/access-(HSF), SpliceAI (https://spliceailookup.broadinstitute.org), ESEfinder 3.0 (https://esefinder.ahc.umn.edu/cgi-bin/tools/ESE3/esefinder.cgi) in the human genome database to predict the pathogenicity of the mutations.

### 2.4 Minigene experiment

#### 2.4.1 Construction of recombinant vector

**FIGURE 1:**
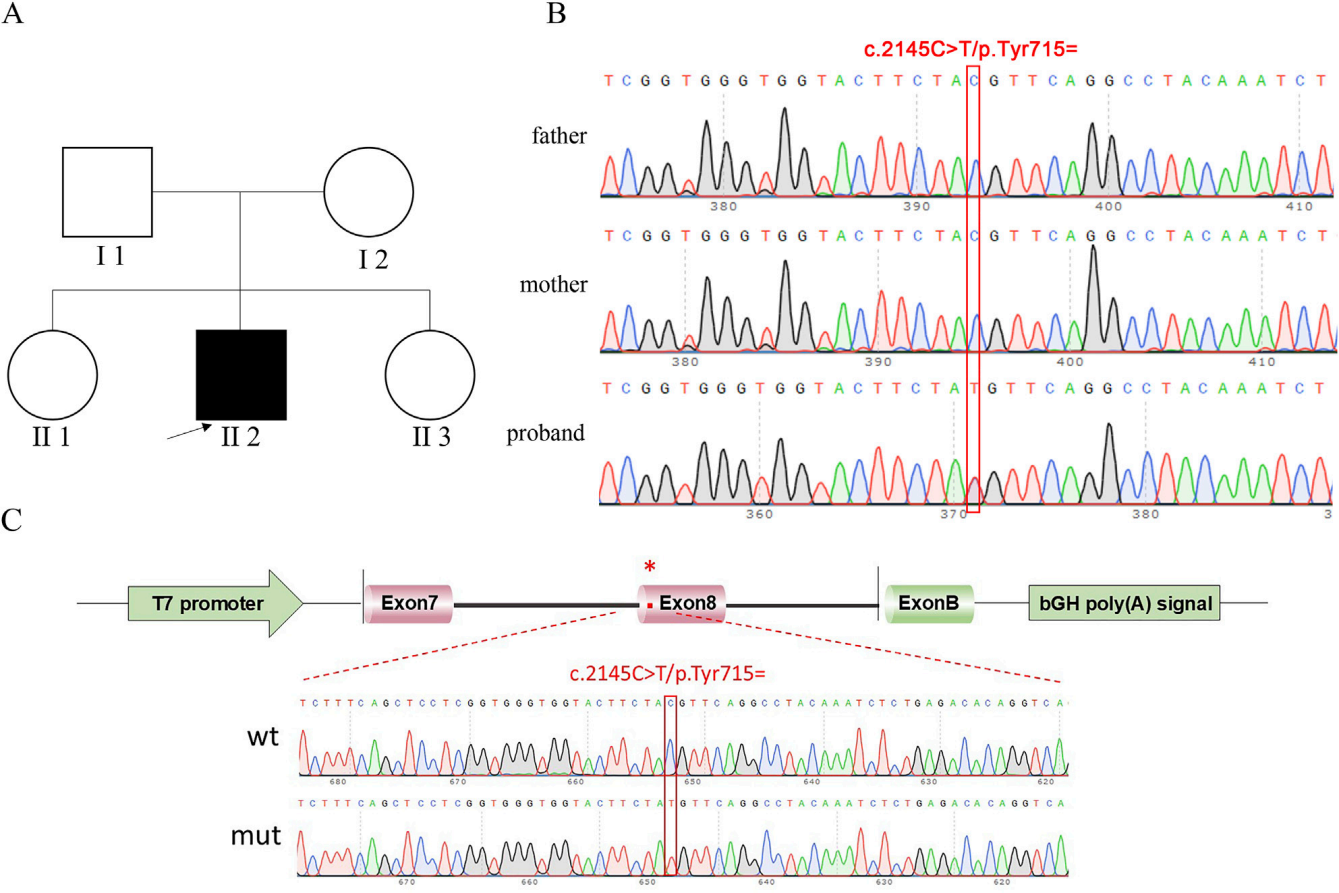
Pedigree of proband and sequencing result. **(A)** Pedigree of proband. II 2, proband (study subjects); I 1, proband’s father; I 2, proband’s mother; black represents diseased; white represents no clinical phenotype. **(B)** Sanger sequencing results of the proband, father, and mother. **(C)** Vector and fragment construction schematic diagram and minigene construction sequencing diagram. wt means wild-type, mut means mutant; red * indicates mutation location.

The ATP7B-wt/mut sequences were amplified using nested PCR. In the first round of PCR, genomic DNA extracted from a healthy individual was used as the template with the primers ATP7B-50453-F and ATP7B-53808-R (30 cycles). The second round of nested PCR utilized the first-round product as the template with the nested primers ATP7B-50730-F and ATP7B-53545-R (30 cycles). The wild-type fragment was amplified from the second-round PCR product using primers pcMINI-N-ATP7B-KpnI-F and pcMINI-N-ATP7B-EcoRI-R, whereas the mutant fragment was generated *via* site-directed mutagenesis (primers listed in Table 1). For recombinant vector construction, the amplified fragments and pcMINI-N vector were digested with restriction enzymes KpnI and EcoRI. The digested products were purified and ligated in a 10-μL reaction mixture (1 μL 10× ligase buffer, 6 μL wild-type/mutant fragment, 2.5 μL digested vector, and 0.5 μL T4 DNA ligase). The minigene pcMINI-N-ATP7B-wt/mut was constructed by inserting the exon 7 (175 bp)–intron 7 (1,603 bp)–exon 8 (234 bp)–partial intron 8 (253 bp) fragment into the pcMINI-N plasmid vector. The schematic diagram of vector-fragment assembly and sequencing verification of the minigene are shown in Figure 1. After confirming the correct sequences by Sanger sequencing, the constructs were transfected into cells.

**TABLE 1:** PCR amplification primer sequence.

Primer name	Sequence
*ATP7B*⁃50453-F	5′-GGA​TGC​TGC​AGC​TAA​CAT​GA⁃3′
*ATP7B*⁃53808-R	5'⁃GTC​CTC​ACC​AAG​GGT​CAC​AA-3′
*ATP7B⁃*50730-F	5′-CTC​ACT​TGC​CTC​ACC​CGT​AA⁃3′
*ATP7B⁃*53545-R	5′-AGT​GAT​GCA​CAG​TGC​CTG​TG⁃3′
pcMINI⁃N⁃*ATP7B*⁃KpnI-F	5′-GCT​TGG​TAC​CAT​GCA​GTG​GAA​GAA​GTC​TTT​CCT​GT⁃3′
pcMINI⁃N⁃*ATP7B*⁃EcoRI-R	5′-TGC​AGA​ATT​CGG​CCA​GGT​TTC​TTT​AGT​TTA⁃3′
*ATP7B⁃*MUT-F	5′-TGG​GTG​GTA​CTT​CTA​TGT​TCA​GGC​CTA​CAA​A⁃3′
*ATP7B⁃*MUT-R	5'⁃TTT​GTA​GGC​CTG​AAC​ATA​GAA​GTA​CCA​CCC​A-3′
pcMINI-N-F	5′-CTA​GAG​AAC​CCA​CTG​CTT​AC-3′
pcMINI-N-R	5′-GCC​CTC​TAG​ACT​GGT​CAT​TCC​GGC​TC-3′
Actin-F	5′-TGA​CGT​GGA​CAT​CCG​CAA​AG-3′
Actin-R	5′-CTG​GAA​GGT​GGA​CAG​CGA​GG-3′

#### 2.4.2 Cell transfection

The minigene recombinant plasmid was transferred into HeLa and 293T cell lines for replication and expression by transient transfection. One day before transfection, both cell lines (293T and HeLa) were trypsinized, passaged, and seeded into 12-well plates. Each well was filled with 1 mL of antibiotic-free medium supplemented with 10% FBS, followed by adding 40 μL of cell suspension. Microscopic observation confirmed a cell density of approximately 50% confluence. The culture dish was gently swirled to ensure even cell distribution and incubated in a cell culture incubator. The required amounts of OPTI-MEM serum-free medium, DNA, and liposome transfection reagent for the 12-well plate were calculated. The pre-incubated DNA–liposome mixture was added to each well and gently mixed, and the plate was returned to the incubator. After 48 h, the culture medium was discarded, and the residual medium was removed by washing twice with PBS buffer. Subsequently, 1 mL of trypsin was added to each well to fully contact the cells. After removing excess trypsin, the plate was incubated for 2–5 min to allow cell detachment. Once cells naturally detached as single-cell suspensions, they were transferred to 1.5 mL EP tubes, centrifuged at 12,000 rpm for 5 s, and the supernatant was discarded. Finally, 1 mL of TRIzol reagent was added for further processing.

#### 2.4.3 RNA extraction

RNA was extracted from cells by the TRIzol method. The methods for obtaining RNA were as follows. The cell samples added with TRIzol reagent were placed at 4°C for 10 min to fully lyse the cells. After 10 min, it was centrifuged at 4°C at 12,000 r/min for 5 min, and the supernatant was transferred to the new EP tube. An amount of 200 μL of chloroform was added to the EP tube, and it was centrifuged at 4°C at12,000 r/min for 15 min after shock. An amount of 400 μL supernatant was taken into a new EP tube, 400 μL isopropyl alcohol was added, and it was placed at −20°C for 10 min. After 10 min, the supernatant was removed by centrifugation at 4°C at 12,000 r/min for 10 min. The precipitation was washed with 1 mL of freshly prepared 75% ethanol and centrifuged for 5 min at 7,000 r/min at 4°C. The supernatant was discarded, the excess liquid was sucked with the tip of the gun, the sample outlet was put on a super-clean table for 3–5 min to dry, 20 μL DEPC water was used to dissolve the precipitation, it was gently blown with a pipette, and the part of the precipitation attached to the tube wall was rinsed to the bottom of the centrifuge tube. The concentration of the product was determined by an RNA concentration purity meter, and the RNA with the absorbance value between 1.8 and 2.0 was stored at 4°C for use.

#### 2.4.4 Reverse transcriptional reaction

The cDNA of the ATP7B gene was obtained by RT-PCR reverse transcription of the RNA of the ATP7B gene, and PCR amplification was performed on the cDNA of the ATP7B gene using Actin-F and Actin-R as primers (the primer sequences are shown in Table 1) to observe whether the internal reference gene amplification was normal. PCR amplification of the cDNA of the ATP7B gene was performed using two primers: pcMINI-N-F and pcMINI-N-R (the primer sequences are shown in Table 1). The PCR amplification product was subjected to agarose gel electrophoresis. If multiple bands appeared, TA cloning was performed, and the monoclonal bacterial solution connected to the T carrier was tested. Sanger sequencing was used to confirm the shear mode of each band.

The TA clone connection system consisted of the following: DNA fragment 8 μL, T-vector 1 μL, enhancer 1 μL, and connection at 22°C for 30 min.

The reverse transcription reaction system consisted of the following: after removal of residual genomic DNA, 10 μL 2× HifairTMⅡ SuperMix plus was added into the reaction tube at 37°C for 2 min and at 55°C for 15 min. Reverse transcription reaction was performed at 85°C for 5 min. The reaction products were stored at 4°C.

## 3 Results

### 3.1 ATP7B gene whole-exome sequencing and Sanger sequencing verification results

In this experiment, WES sequencing technology was used to detect 21 exons of ATP7B gene in samples to be tested, and the DNA of the family was used as a control to determine whether the progenitor gene had mutations. According to the family tree of the proband (Figure 1), the whole exon sequencing of the ATP7B gene, and the verification results of the Sanger sequencing, it was clear whether the object of this study had suspicious gene mutations. Primers were designed for suspected pathogenic sites, and minigene experiments were performed.

The progenitor had a frameshift mutation in the ATP7B gene of c.2304 dupC (p. M. 769HISFS *26) that had been identified as pathogenic. At the same time, the study identified a heterozygous synonymous mutation c.2145C>T (p. Tyr715 =), where nucleotide C was substituted by T at position 2,145 of the ATP7B gene. This mutation was a synonymous variant that did not alter the amino acid sequence and was identified as a candidate pathogenic site in this study.

### 3.2 Bioinformatics prediction

**FIGURE 2:**
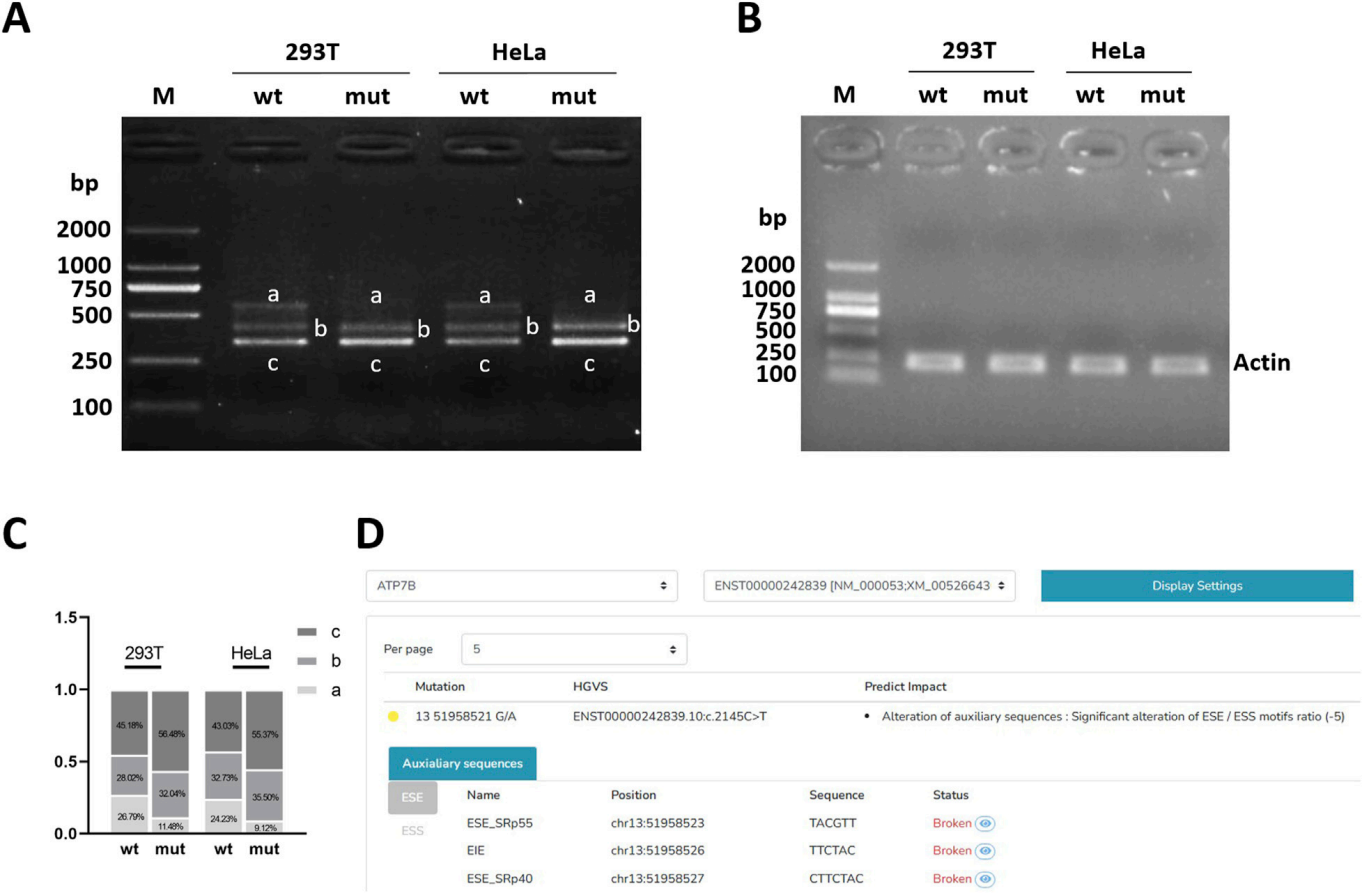
Electrophoretic detection of RT-PCR products. **(A)** Electrophoresis results of target gene RT-PCR products. **(B)** Electrophoresis results of internal reference gene actin RT-PCR product. **(C)** Grayscale statistical histogram of electrophoresis bands of target gene RT-PCR products. **(D)** HSF predicts the effect of mutation on splicing. Note: M, marker; mut, the mutation bands of post-transcriptional exon c.2145C>T(p. Tyr =); wt, the wild-type target bands.

HSF suggested that c.2145C>T (p. Tyr715 =) mutation may affect nearby shear auxiliary sites Figure 2D. ESEfinder 3.0 suggests that the mutation may affect the binding of ESE to SRp40 and SRp55 proteins, thus affecting shear. SpliceAI suggests that mutations may affect clipping. Mutations are not recorded in the gnomAD database. Mutation taster showed that the mutation was a “pathogenic mutation,” which may have an effect on shear, and the specific results are shown in Table 2.

**TABLE 2:** Bioinformatics predictions.

Gene	Mutation location	ESEfinder 3.0	SpliceAI	HSF	gnomAD	Mutation taster
*ATP7B*	c.2145C>T(p.Tyr715 =)	Potentially affect	Potentially affect	Potentially affect	Not included	Pathogenic mutation

### 3.3 Electrophoretic detection of RT-PCR products

After agarose gel electrophoresis of the cDNA amplification product, the bands of wild-type (WT) and mutant (MUT) were examined in a visible ultraviolet detector in a UV chamber. The results of reference gene amplification showed that the amplified bands were single and the size was in line with expectations (Figure 2B). It can be roughly observed that both wild-type (WT) and mutant (MUT) have three bands, with two bands ranging from 250 to 500 bp and one band ranging from 500 to 750 bp, respectively, and one band ranging from 500 to 750 bp of mutant (MUT) is almost absent compared with wild-type (WT) (Figure 2A). After gray scanning of the wild-type and mutant bands, it was found that in 293T cells, wild-type band a accounted for 26.79% and mutant band a accounted for 11.48%. In HeLa cells, wild-type band a accounted for 24.23% and mutant band a accounted for 9.12%. The proportion of mutant band a (normal cut band) in both cells was significantly reduced (Figure 2C).

### 3.4 Sequencing results of cDNA-T carrier solution

The difference between wild-type (WT) and mutant-type (MUT) bands was checked in the UV chamber visible UV detector; after purification of the PCR product, TA cloning, and connection of each band to the T carrier, bacterial liquid-cDNA-T with different size bands of wild-type and bacterial liquid-cDNA-T with different size bands of mutant type were selected for Sanger sequencing, and the results were as follows.

#### 3.4.1 The splicing pattern of the wild-type band

**FIGURE 3:**
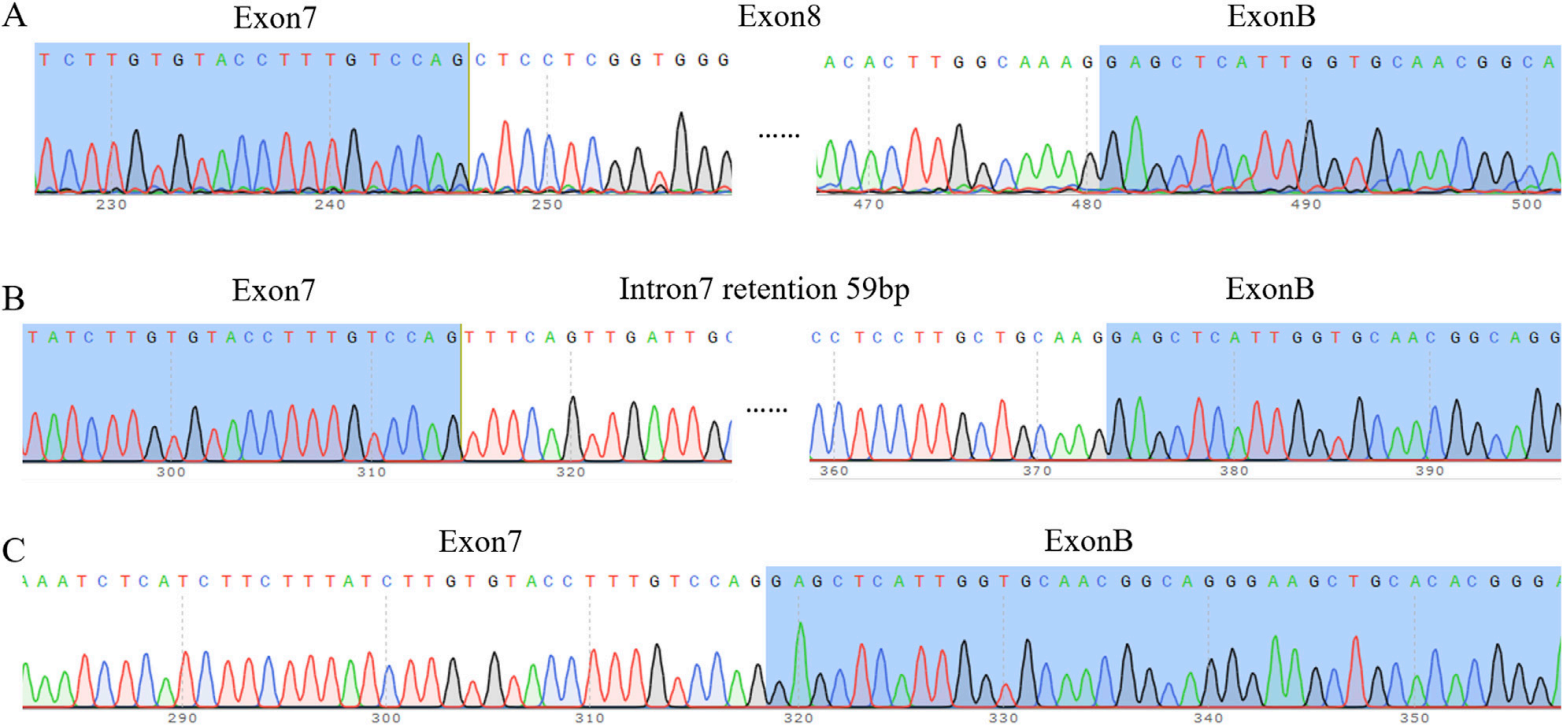
Sequencing results. **(A)** Sequencing results of wild-type band a and mutant band a **(B)** Sequencing results of wild-type band b and mutant band b. Note: intron 7 internal retention 59 bp, exon 8 deletion. **(C)** Sequencing results of wild-type band c and mutant band c. Note: exon 8 deletion.

Wild-type strip a was sheared as exon 7 (175 bp)–exon 8 (234 bp)–exon B (57 bp), with a total of 575 bp, which was a normally sheared mature mRNA (Figure 3A). Wild-type strip b was sheared as exon 7 (175 bp)–∇intron 7 (59 bp)–exon B (57 bp), 400 bp in total, with 59 bp stranded on the left side of intron 7 and exon 8 jumping, which was an abnormally sheared mRNA (Figure 3B). The wild-type strip c was sheared as exon 7 (175 bp)–exon B (57 bp), with exon 8 missing, for a total of 341 bp, which is an abnormally sheared RNA (Figure 3C).

#### 3.4.2 The splicing pattern of the mutant band

Mutant band a was sheared as exon 7 (175 bp)–exon 8 (234 bp)–exon B (57 bp), and it was normally sheared like wild-type band a. Mutant stripe b was sheared in exon 7 (175 bp)–∇intron 7 (59 bp)–exon B (57 bp), the same way as wild-type stripe b was sheared. The mutant strip c shear pattern was exon 7 (175 bp)–exon B (57 bp) with exon 8 deletion, the same as the wild-type strip c shear pattern.

## 4 Discussion

Hepatolenticular degeneration is an autosomal recessive disease with ATP7B as the pathogenic gene. The occurrence of missense mutations, nonsense mutations, deletions, and insertions in the ATP7B gene (Wang et al., 2022) may be one of the contributing factors to hepatic lenticular degeneration. Therefore, the current diagnosis is mainly based on a combination of ATP7B gene testing and the patient’s clinical symptoms, and for study subjects with a family history, the only diagnostic modality before the onset of symptoms is ATP7B gene sequencing (Tasmeen et al., 2022). Verification of the effect of mutations in the ATP7B gene on pathogenicity can not only effectively improve the diagnosis of hepatolenticular degeneration but also provide theoretical support and research ideas for the study of other genetic diseases. In this study, a synonym mutation c.2145C>T (p. Tyr715 =) was found in the ATP7B gene by total external sequencing in the proband, but the pathogenicity of this synonym mutation had not been confirmed.

With the development of second-generation DNA sequencing technology, the pathogenicity of synonymous mutations has been recognized by most scholars (Panzer et al., 2022). Studies have found that synonymous mutations can lead to changes in the splicing mode of precursor mRNA, and mis-splicing of precursor mRNA is a very important pathogenic mechanism of recessive genetic diseases (Wen and Li, 2001). The normal splicing process involves the protein component of the spliceosome acting as an enzyme to remove the introns within the spliceosome and ligate the exons together (Chu and Xu, 2023). After splicing, the mRNA could be translated into proteins. The prior witnesses in this study had the identified pathogenic c.2304dupC (p. Met769Hisfs*26) code-shift mutation in the ATP7B gene and the c.2145C>T (p. Tyr715 =) synonymous mutation, which bioinformatics analysis suggested may be pathogenic. As there were no previous reports of pathogenicity associated with this synonymous mutation, bioinformatics prediction was used to first predict the effect of the mutant on the precursor mRNA splicing mode. Bioinformatics analysis by HSF, SpliceAI, and ESEfinder 3.0 suggested that the mutation may affect the normal splicing of the precursor mRNA, thus affecting the normal function of the gene. This was consistent with the results predicted by previous scholars for synonymous mutations at different sites of ATP7B (Xu et al., 2023), which may be the pathogenic mechanism of hepatolenticular degeneration.

In order to further verify the effect of the synonym mutation on normal splicing *in vitro*, the minigene experiment was used in this study to verify the effect of the synonym mutation on splicing mode in HeLa cells and 293T cells. The minigene electrophoresis results showed that the synonymous mutation c.2145C>T (p. Tyr715 =) on the ATP7B gene more greatly reduced the abundance of normally spliced mRNAs compared to the ATP7B in wild type, which may be the mechanism responsible for the pathogenicity of this lineage. Sequencing of RT-PCR products after T-loading showed that there were three types of splicing in both wild type and mutant, where one was normal splicing and the remaining two were abnormal splicing. The abnormal banding patterns of wild type and mutant type include (1) 59 bp retention on the left side of intron 7, and exon 8 was skipped; (2) exon 8 was lacking. In the electrophoretic map, the mutant 566 bp band was darker than the wild type, indicating that the wild-type band had a higher proportion of normal splicing, whereas the mutant band had a higher proportion of abnormal splicing and a lower proportion of normal splicing. This may be one of the mechanisms that leads to hepatolenticular degeneration.

SRp40 and SRp55 proteins belong to the family of SR proteins, which are RNA-binding proteins that function mainly in RNA splicing. SR proteins are characterized by the presence of C-terminal structural domains (RS structural domains) and N-terminal RNA recognition structural domains (RRM structural domains) rich in arginine (R) and serine (S) amino acid sequences, and they are known as constitutive and selective splicing regulators (Jeong, 2017). The synonymous mutation c.2145C>T (p. Tyr715 =) on the ATP7B gene affected the binding of the exonic splicing enhancer to the RNA-binding proteins, which resulted in the inhibition of the splicing enhancer function and affected the normal splicing of the precursor mRNA. This resulted in a decrease in the amount of mRNA required for normal splicing, which ultimately affected the amount of protein expressed in the mutant phenotype. Roy S et al. indicated that the pathogenicity of the ATP7B missense mutation p.G1101R may be due to a combination of factors such as its impaired function, retention in the endoplasmic reticulum, and low expression levels (Roy et al., 2020). Daniele Merico et al. demonstrated by constructing gene-edited HepG2 cells that c.1934T>G causes Wilson’s disease primarily by altering splicing and reducing ATP7B expression (Merico et al., 2020). These experimental results demonstrated that reduced expression of ATP7B may contribute to the pathogenesis.

In this family, individuals simultaneously carry the c.2304dupC (p. Met769Hisfs*26) frame shift mutation, which leads to either NMD (nonsense-mediated mRNA decay) degradation or the formation of truncated proteins, resulting in functional defects of the ATP7B protein. Based on the ACMG guideline rating, the mutation was identified as LP (likely pathology) suspected pathogenicity. This mutation, together with c.2145C>T (p. Tyr715 =), constitutes a compound heterozygous mutation in the prior witness, originating from each parent. Because Wilson’s disease was a treatable autosomal recessive disorder, this trans-double mutation was consistent with the inheritance of AR genetic disorders.

In summary, this experiment, based on minigene technology, confirmed that the mutation c.2145C>T (p. Tyr715 =) in the locus on exon 8 of the ATP7B gene could lead to the abnormal splicing of the precursor mRNA. This mutation leads to a decrease in the splicing abundance of transcripts from the normal stripe of mRNA required for splicing of the precursor mRNA, thus affecting the expression of subsequent proteins. This further confirms that the mutation at this site in the ATP7B gene indeed causes splicing abnormalities, leading to a genetic disease. This provides theoretical support for diagnosing and preventing the occurrence of related diseases, expands the spectrum of pathogenic mutations in the ATP7B gene, and offers theoretical support and research ideas for future studies on related genetic diseases.

## Data Availability

The original contributions presented in the study are included in the article/supplementary material; further inquiries can be directed to the corresponding authors.

## References

[B1] Chu Y. R. Xu S. Q. (2023). Research progress of alternative splicing in autoimmune diseases. Chin. J. General Pract. 21 (7), 1211–1214. 10.16766/j.cnki.issn.1674-4152.003086

[B2] European Association for Study of Liver (2012). EASL clinical practice guidelines: Wilson's disease. J. Hepatol. 56 (3), 671–685. 10.1016/j.jhep.2011.11.007 22340672

[B3] Hartwig C. Zlatic S. A. Wallin M. Vrailas-Mortimer A. Fahrni C. J. Faundez V. (2019). Trafficking mechanisms of P-type ATPase copper transporters. Curr. Opin. Cell Biol. 59, 24–33. 10.1016/j.ceb.2019.02.009 30928671 PMC6726579

[B4] Huang Y. Liu Z. F. (2019). Research advance of *ATP7B* gene mutation in hepatolenticular degeneration. Med. Recapitulate 25 (9), 1717–1721. 10.3969/j.issn.1006-2084.2019.09.010

[B5] Jeong S. (2017). SR proteins: binders, regulators, and connectors of RNA. Mol. Cells 40 (1), 1–9. 10.14348/molcells.2017.2319 28152302 PMC5303883

[B6] Kodama H. Fujisawa C. Bhadhprasit W. (2012). Inherited copper transport disorders: biochemical mechanisms, diagnosis, and treatment. Curr. Drug Metab. 13 (3), 237–250. 10.2174/138920012799320455 21838703 PMC3290776

[B7] Lao T. D. Le T. A. H. (2024). Systematic analysis and insights into the mutation spectrum and ethnic differences in ATP7B mutations associated with Wilson disease. Biomark. Insights 19, 11772719241297169. 10.1177/11772719241297169 39502306 PMC11536366

[B8] Love S. L. Emerson J. D. Koide K. Hoskins A. A. (2023). Pre-mRNA splicing associated diseases and therapies. RNA Biol. 20 (1), 525–538. 10.1080/15476286.2023.2239601 37528617 PMC10399480

[B9] Merico D. Spickett C. O'Hara M. Kakaradov B. Deshwar A. G. Fradkin P. (2020). ATP7B variant c.1934T>G p. Met645Arg causes Wilson disease by promoting exon 6 skipping. NPJ Genom Med. 5, 16. 10.1038/s41525-020-0123-6 32284880 PMC7142117

[B10] Panzer M. Viveiros A. Schaefer B. Baumgartner N. Seppi K. Djamshidian A. (2022). Synonymous mutation in adenosine triphosphatase copper-transporting beta causes enhanced exon skipping in Wilson disease. Hepatol. Commun. 6 (7), 1611–1619. 10.1002/hep4.1922 35271763 PMC9234614

[B11] Roy S. McCann C. J. Ralle M. Ray K. Ray J. Lutsenko S. (2020). Analysis of Wilson disease mutations revealed that interactions between different ATP7B mutants modify their properties. Sci. Rep. 10 (1), 13487. 10.1038/s41598-020-70366-7 32778786 PMC7418023

[B12] Tasmeen R. Karim A. S. M. B. Banu L. A. Hossain E. Rokunuzzaman M. Majumder W. (2022). Mutational analysis of exon 8 and exon 14 of ATP7B gene in Bangladeshi children with Wilson disease. Indian J. Gastroenterology 41 (5), 456–464. 10.1007/s12664-022-01276-x 36308701

[B13] Wang J. W. Chang L. Gu T. Jia Z Zhang A Liu Z (2022). A case of missense and synonymous mutations in *ATP7B* gene in a family with hepatolenticular degeneration. Ningxia Med. J. 44 (5), 385–387. 10.13621/j.1001-5949.2022.05.0385

[B14] Wang X. D. Guo L. R. Zhang S. H. Chen Y. Chen Y. T. Zheng B. (2019). Copper sulfide facilitates hepatobiliary clearance of gold nanoparticles through the copper-transporting ATPase ATP7B. ACS Nano 13 (5), 5720–5730. 10.1021/acsnano.9b01154 30973228 PMC8325778

[B15] Wen F. Li Y. D. (2001). A bioinformatic analysis of alternatively spliced genes of human. Acta Electron. Sin. 29 (S1), 1735–1739. 10.3321/j.issn:0372-2112.2001.z1.002

[B16] Xu W. Q. Wang R. M. Dong Y. Wu Z. Y. (2023). Pathogenicity of intronic and synonymous variants of *ATP7B* in Wilson disease. J. Mol. Diagnostics 25 (1), 57–67. 10.1016/j.jmoldx.2022.10.002 36343861

